# Transcranial Direct Current Stimulation of the Dorsolateral Prefrontal Cortex Modulates Cognitive Function Related to Motor Execution During Sequential Task: A Randomized Control Study

**DOI:** 10.3389/fnhum.2022.890963

**Published:** 2022-06-14

**Authors:** Satoshi Yamamoto, Daisuke Ishii, Kiyoshige Ishibashi, Yutaka Kohno

**Affiliations:** ^1^Department of Physical Therapy, Ibaraki Prefectural University of Health Sciences, Ami, Japan; ^2^Center for Medical Sciences, Ibaraki Prefectural University of Health Sciences, Ami, Japan; ^3^Department of Cognitive Behavioral Physiology, Chiba University Graduate School of Medicine, Chiba, Japan; ^4^Department of Physical Therapy, Ibaraki Prefectural University of Health Sciences Hospital, Ami, Japan

**Keywords:** sequential task, dorsolateral prefrontal cortex, transcranial direct current stimulation, Stroop task, supervisory attention system

## Abstract

In daily life, we perform a variety of sequential tasks while making cognitive decisions to achieve behavioral goals. If transcranial direct current electrical stimulation (tDCS) can be used to modulate cognitive functions involved in motor execution, it may provide a new rehabilitation method. In the present study, we constructed a new task in which cognitive decisions are reflected in motor actions and investigated whether the performance of the task can be improved by tDCS of the left dorsolateral prefrontal cortex (DLPFC). Forty healthy participants were randomly assigned to a real or sham tDCS group. The anode electrode was placed at F3 (left DLPFC), and the cathode electrode was positioned in the contralateral supraorbital area. Participants underwent one session of tDCS (1.5 mA, 20 min) and a sequential non-dominant hand task was performed for nine trials before and after tDCS. The task consisted of S1 (a manual dexterity task) and S2 (a manual dexterity task requiring a decision). The results showed the S2 trajectory length was significantly shorter after real tDCS than after sham tDCS (*p* = 0.017), though the S1 trajectory length was not significant. These results suggest that a single tDCS session of the left DLPFC can improve the performance of cognitive tasks complementary to motor execution, but not on dexterity tasks. By elucidating the modulating effect of tDCS on cognitive functions related to motor execution, these results may be used to improve the performance of rehabilitation patients in the future.

## Introduction

Tasks in daily life typically involve a series of actions performed to achieve a goal. Sequential tasks require decision-making, such as selecting the next action based on the perception of the situation and deciding how to execute that action. Executive function involves multiple brain functions and regions (Salehinejad et al., 2021a). The frontal lobe, particularly the prefrontal cortex (PFC) is activated during cognitive and decision-making processes (Miller and Cohen, 2001). Specifically, the PFC is associated with most sensory systems, cortical areas, and subcortical structures involved in behavior, motor responses, memory, and emotion (Miller and Cohen, 2001). The lateral PFC is involved in logical top-down cognitive processes (Chan et al., 2008) and functions in an emotionally neutral context (Zelazo and Carlson, 2012). Moreover, the dorsolateral prefrontal cortex (DLPFC) is involved in working memory, attention, executive function, dual tasks, and other related areas (Adleman et al., 2002; Badre and Wagner, 2004; Harty et al., 2014; Huang et al., 2017; Brzezicka et al., 2019; Jamali et al., 2019; Panikratova et al., 2020). Imaging studies using functional magnetic resonance imaging have also implicated the DLPFC in bimanual coordination tasks (Beets et al., 2015) and explicit sequence learning (Schendan et al., 2003; Meehan et al., 2011). These studies indicate that the DLPFC is not only involved in cognitive function, but also in complex motor tasks and motor learning.

Transcranial direct current stimulation (tDCS) noninvasively modulates cortical activity using an electric current delivered from the scalp surface (Polania et al., 2018). Previous research has demonstrated that anodal tDCS (atDCS) activates, whereas cathodal tDCS inhibits the cortical activity (Nitsche and Paulus, 2000, 2001). The effect of atDCS on the DLPFC on reaction time in healthy participants has been reported to significantly decrease reaction time during cognitive tasks (Dedoncker et al., 2016). Furthermore, a meta-analysis of atDCS on executive function found that a tDCS on the DLPFC improved performance on an update task (Imburgio and Orr, 2018). Studies with anodal stimulation on the left DLPFC have reported faster reaction times on the Stroop task (Loftus et al., 2015) and improved performance on the emotional working memory task (Vanderhasselt et al., 2013). These previous studies indicate that atDCS on the left DLPFC can radically improve cognitive function.

The effects of atDCS on motor function and learning have been reported to differ depending on the site of electrode placement. The effect of a tDCS on motor function has been reported to increase maximal voluntary muscle contraction and endurance (Lattari et al., 2018), and improve motor function in stroke patients using electrodes on M1 (Hummel and Cohen, 2005). Conversely, atDCS with electrodes placed on the left DLPFC does not affect the performance of bimanual coordinated movements (Vancleef et al., 2016). Moreover, the effect of atDCS on motor learning, which is more complex than motor function and involves multiple brain regions, has been found to improvements in the learning performance of a serial task when electrodes are placed on M1 during a serial reaction time task (SRRT; Hashemirad et al., 2016); however, atDCS stimulation on the left DLPFC improved neither SRTT performance (Nitsche et al., 2003) nor memory learnings (Hammer et al., 2011). These reports suggest that atDCS on the left DLPFC lacks a modulation effect on motor function and learning.

Previous studies have shown that atDCS on the left DLPFC improved cognitive function but not motor function or learning; however, the effects of modulation using atDCS by performing a single motor task or a motor learning task have been evaluated. Few studies have performed cognitive and motor tasks as a series of movements or as dual tasks performed simultaneously, rather than as a single task. In a previous study, a cognitive and manual dual task was performed during tDCS with an anode on the left DLPFC. The results showed that the number of subtractions in the Serial Seven Subtraction Test significantly increased during tDCS stimulation compared to that before tDCS stimulation (Ljubisavljevic et al., 2019). This study suggests that atDCS to the left DLPFC modulates cognitive rather than motor function in a dual task setting. The Serial Seven Subtraction Test used in this study was a cognitive task that was both inhibitory and competitive with movement; however, in daily life, we routinely adjust our movements because of cognitive decisions. Thus, the relationship between cognition and movement is complementary rather than competitive. Currently, the modulation effect of atDCS on the left DLPFC in tasks in which cognition and movement are complementary remains unclear.

The current study aimed to investigate whether atDCS on the left DLPFC improves performance in a cognitive task complementary to motor execution. We hypothesized that since atDCS on the left DLPFC improves cognitive function in a task in which motor and cognitive functions are competitive (Ljubisavljevic et al., 2019), tDCS will similarly improve task performance in a cognitive task complementary to the motor execution.

## Materials and Methods

### Participants

Thirty-six males and four females (mean age: 25.6 years; range: 18–44 years) participated in the current study. The sample size was calculated using G*Power 3.1.9.7 for Windows (Faul et al., 2007, 2009). There were 20 participants in each group (input parameters were as follows: effect size = 0.33, α error probability = 0.05, and power = 0.8).

None of the participants had a history of neurological or psychiatric disease, or any condition associated with somatosensory abnormalities, as determined by a non-structured interview. All participants understood the instructions for the experimental tasks and provided written informed consent to participate. The present study was performed in accordance with the principles of the Declaration of Helsinki. The study protocol was approved by the Ethics Committee of the Ibaraki Prefectural University of Health Sciences (approval no. 960).

### Experimental Design and Procedure

The participants underwent one tDCS session (anodal or sham). These self-paced tasks were performed by the participant for 20 min during the tDCS session. The evaluation of learning enhancement by tDCS on the tasks was conducted in nine trials of the same tasks before and after tDCS. A trained male examiner conducted all experiments. Each session lasted approximately 1 h. The Edinburgh Handedness Inventory was used to evaluate the participants’ handedness (Oldfield, 1971). The time of day when the tDCS session was conducted was recorded as taking place in the morning or afternoon.

### tDCS

We conducted a double-blind, randomized controlled study of anodal or sham tDCS. Participants were randomly assigned to the real or sham tDCS groups and were not informed which group they were allocated to. To blind the experimenter to the experimental conditions, another researcher randomly selected a 5-digit experimental code from the list and determined the experimental conditions for the participants. The tDCS was delivered by a pair of saline-soaked sponge (35 cm^2^: 7 × 5 cm) surface electrodes (neuroConn, IImenau, Germany) for 20 min at 1.5 mA current intensity. Active stimulation was performed with an anodal electrode placed on the participant’s left DLPFC, represented by the F3 electrodeposition on the scalp, according to the 10/20 EEG system (Santos Ferreira et al., 2019). The cathodal electrode was placed on the contralateral supraorbital area as in previous studies (Heeren et al., 2015). For the sham condition, stimulation was applied for only the first 30 s to prevent the participant from determining their group. After the experiment, the experimenter verbally asked the participants whether they thought the experimental conditions were real or sham. Their responses were recorded in the experiment notebook.

### Sequential Task

**Figure 1:**
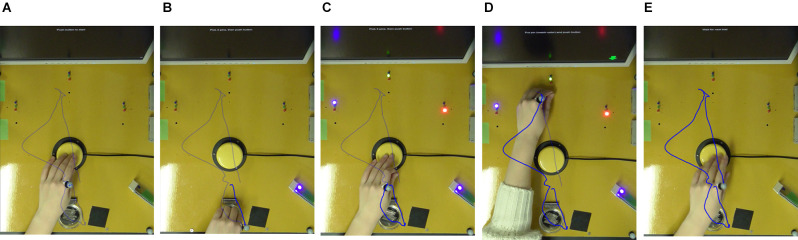
Sequential task. The sequential task was performed as follows. **(A)** Press the switch with the non-dominant hand. **(B)** Pick up three pegs in the peg container. **(C)** After pressing the switch, instructions on where to place the peg are displayed on the monitor. **(D)** After placing the peg, **(E)** press the switch to complete one trial. The trajectory of the metacarpophalangeal joint of the index finger of the non-dominant hand is indicated by a blue line.

Figure 1 shows how the sequential task was conducted. The sequential tasks from A-C include reaching and the manual dexterity task of picking up the pegs (S1). From C-E, the tasks include reaching and the manual dexterity task of putting in the pegs, as well as a modified Stroop task that involves a decision on where to put the pegs (S2). In the modified Stroop task, instructions were given by the color or location of the arrows presented on the monitor. Each hole was illuminated by one of the three LED colors: red, blue, or green. On the display, a red, blue, or green arrow was presented on the monitor on the left, center, or right side. In addition, the monitor showed a condition to either place it in the hole where the LED of the same color as the arrow was illuminated (match-color condition) or in the hole where the arrow was presented (match-place condition).

The sequential and modified Stroop tasks were controlled using MATLAB functions Psychophysics Toolbox Version 3 (Brainard, 1997), and a USB data acquisition device (Labjack U3, LabJack Corporation) was used. The time required to reach S1 and S2 was obtained by measuring the time required to press the switch. The amount of time required to pick up and insert a peg was measured using a photoelectric sensor (NX5-M10RB, Panasonic Co. Ltd.).

The trajectories of infrared reflective markers attached to the metacarpophalangeal joint of the index finger of the non-dominant hand during the S1 and S2 movements were measured (sampling rate: 120 Hz) using a 3D motion analysis system (OptiTrack V120: Trio, Acuity Inc.). The zero point of the coordinates of the system was set as the center point of the switch, which was 15 cm in front of the peg container.

Trials were counted as failures when the participant either failed to take three pegs, dropped a peg before placing it in the target, or placed a peg in the wrong target.

### Statistical Analysis

Statistical analysis was performed with SPSS ver. 28 (SPSS Inc., Chicago, IL, USA). The characteristics of each group were compared using unpaired *t*-tests for age, Edinburgh Handedness Inventory, number of trials during the tDCS session, and number of error trials. For categorical data, such as sex, participants’ awareness of the condition, and session time of day, a chi-squared test was used. The xy component of the trajectory of the reflective marker was used to calculate the trajectory length and the rectangular area, which were analyzed separately for S1 and S2.

Two-way analysis of variance (ANOVA) was performed for trajectory length, rectangular area, reach time, and manual dexterity task time, and multiple comparisons were performed for time (before and after the stimulus) and stimulus (real and sham) factors. Multiple comparisons were performed using the Bonferroni method at a significance level of *p* = 0.05.

## Results

**Figure 2:**
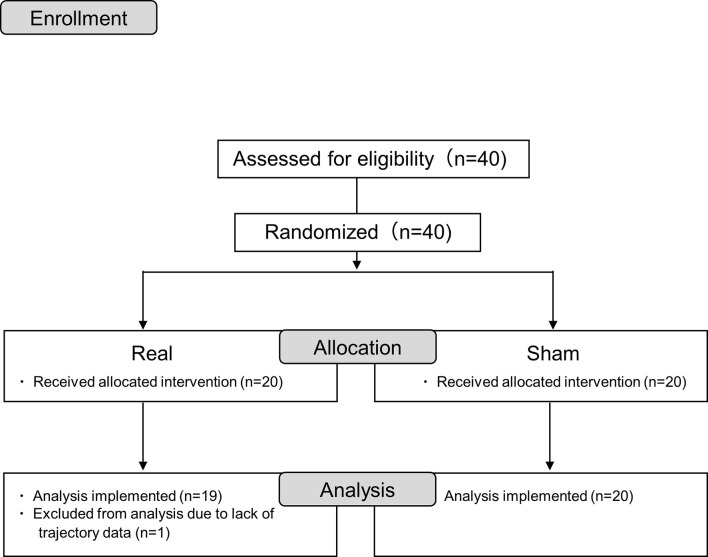
Consort flow diagram.

Forty participants were randomly assigned to the real or sham tDCS groups with 20 participants in each. One participant was excluded due to incomplete data measurement; therefore, the analysis was performed on 19 and 20 participants in the real and sham groups, respectively (Figure 2). All participants in the present study were able to undergo tDCS and pre- and post-session measurements. There were no complaints of side effects from tDCS, which were confirmed verbally after the session.

Table 1 shows the average values of the participants’ characteristics in the real and sham tDCS groups. In terms of age and the Edinburgh Handedness Inventory, the chi-squared and two-sample *t*-tests showed no significant differences, respectively (*χ*^2^ = 0.079, *p* = 0.556; *t* = 0.247, *p* = 0.867). All participants scored at least 50 points on the Edinburgh Handedness Inventory, indicating right-handedness. There was also no significant difference in sex between the groups (*χ*^2^ = 1.004, *p* = 0.316). The tDCS session time of day was not different between groups (*χ*^2^ < 0.001, *p* > 0.999); however, participant awareness of their group was significantly different between groups (*χ*^2^ = 8.313, *p* = 0.004). There was no significant difference in the number of trials during the tDCS session (*t* = 0.458, *p* = 0.729) and that of erroneous trials (*t* = 0.867, *p* = 0.863).

**Table 1:** Average values of the participants’ characteristics in the real and sham tDCS groups.

	Real (n = 19)	Sham (n = 20)	t (df = 37) or χ^2^ (df = 1)	*p*
Age (years)	25.7 ± 6.8	25.6 ± 7.9	0.079	0.556
Edinburgh Handedness Inventory	87.4 ± 16.0	86.2 ± 16.0	0.247	0.867
Gender (M:F)	18:1	17:3	1.004	0.316
Participants’ awareness of the condition (real:sham)	10:9	2:18	8.313	0.004
Number of trials during tDCS session	111.7 ± 13.2	109.8 ± 12.5	0.458	0.729
Number of error trials	8.3 ± 5.1	6.7 ± 6.4	0.867	0.863
Session time of day (AM:PM)	6:13	6:14	<0.001	>0.999

*Notes: data are represented as mean ± SD. Between-group differences are reported with a p-value for the independent samples *t*-test, while for nominal data, the p-value for the χ^2^ statistic is reported*.

The mean sequential task time for each of the nine pre- and post-session times for each participant was as follows: for the pre-session, the real and sham tDCS groups required 8.53 ± 2.11 s and 8.64 ± 1.98 s, respectively. For post-session, the real and sham groups required 6.45 ± 1.20 s and 6.70 ± 0.93 s, respectively. No significant differences were found between groups (pre-session: *t* = 0.159, *p* = 0.874; post-session: *t* = 0.703, *p* = 0.487).

### Effects of tDCS on the Trajectory of the Hand

**Figure 3:**
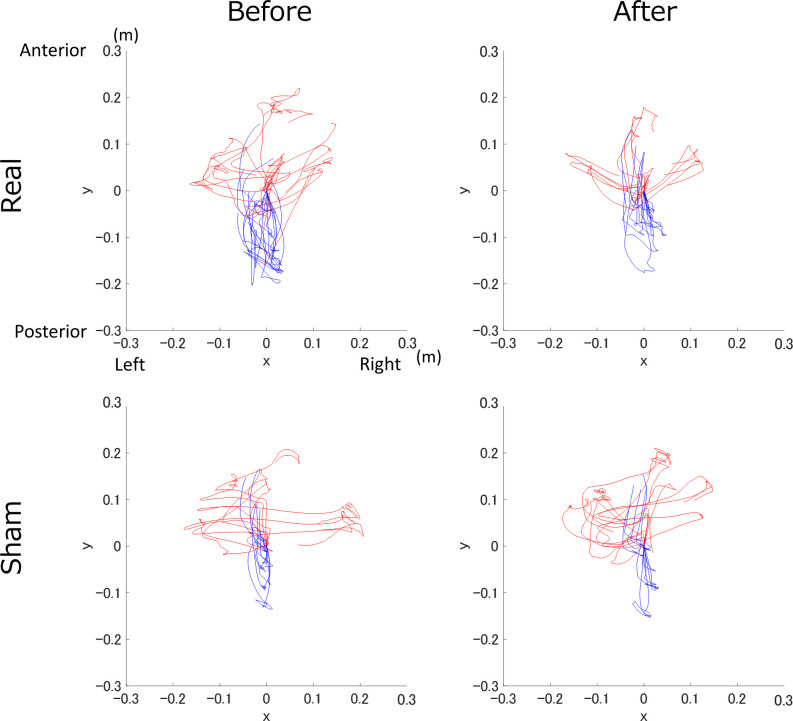
Trajectory example of the real and sham groups before and after transcranial direct current electrical stimulation (tDCS). The top row shows the real tDCS group, the bottom row shows the sham tDCS group, and the columns from left to right are before and after tDCS. The S1 and S2 trajectories are shown in blue and red, respectively.

Trajectory examples of the sequential task were presented to one person in each group (Figure 3).

**Figure 4:**
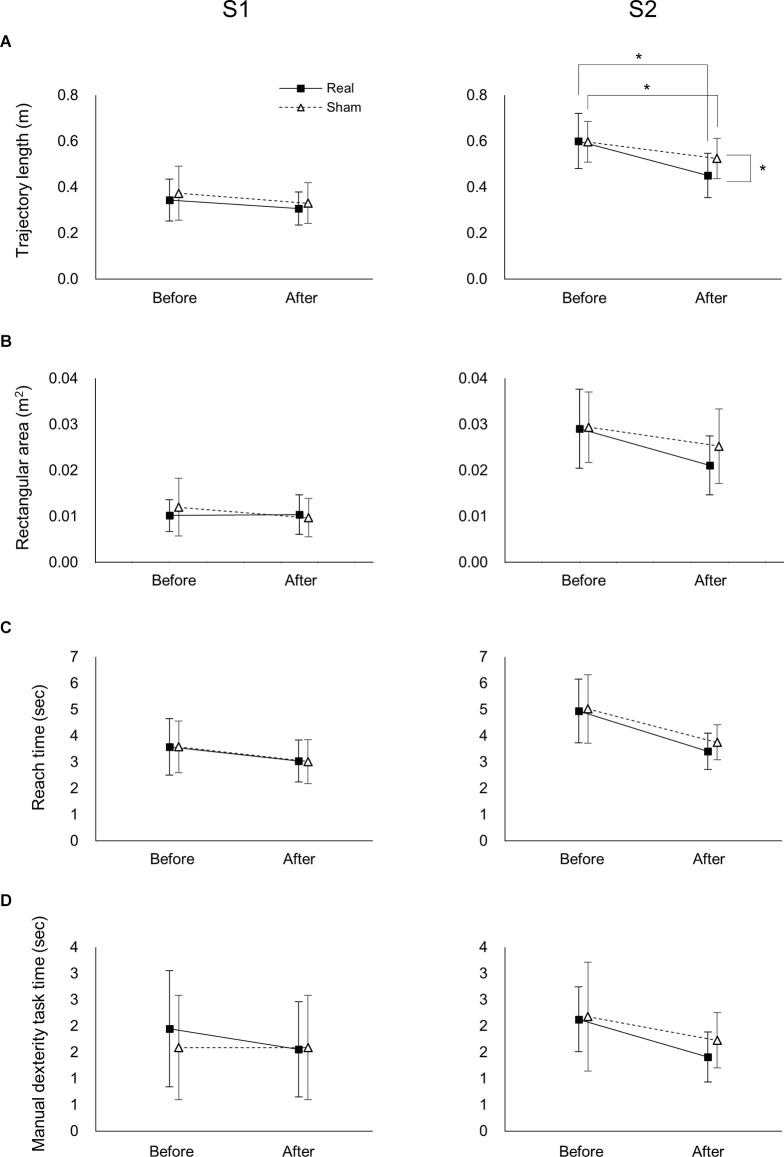
Effects of tDCS on the trajectory, reach time, and manual dexterity task time of the real and sham groups before and after tDCS. Effects of tDCS on trajectory length **(A)**, rectangular area **(B)**, reach time **(C)**, and manipulation time **(D)** on S1 (left panel) and S2 (right panels). Data represent mean ± SD. *Indicates *p* < 0.05.

For the S1 trajectory length, there was a significant main effect of time (*F*_(1,37)_ = 8.763, *p* = 0.005, $\eta_{p}^{2}$ = 0.191), but not stimulation (*F*_(1,37)_ = 0.974, *p* = 0.330, 0$\eta_{p}^{2}$ = 0.026; Figure 4A). Moreover, there was no significant interaction between time and stimulation (*F*_(1,37)_ = 0.068, *p* = 0.795, $\eta_{p}^{2}$ = 0.002).

In the S2 trajectory length, two-way ANOVA showed a significant interaction between time and stimulation (*F*_(1,37)_ = 5.191, *p* = 0.029, $\eta_{p}^{2}$ = 0.123; Figure 4A). A *post-hoc* comparison indicated that the S2 trajectory length after real tDCS was significantly lower than before real tDCS (*p* < 0.001). Similarly, the S2 trajectory length after sham tDCS was significantly shorter than before sham tDCS (*p* = 0.004). Moreover, the S2 trajectory length after the real tDCS was significantly shorter than after the sham tDCS (*p* = 0.017).

In the S1 rectangular area, there was no significant main effect of time or stimulation (*F*_(1,37)_ = 1.418, *p* = 0.241, $\eta_{p}^{2}$ = 0.037; *F*_(1,37)_ = 0.232, *p* = 0.633, $\eta_{p}^{2}$ = 0.006, respectively; Figure 4B). Moreover, there was no significant interaction between time and stimulation (*F*_(1,37)_ = 1.986, *p* = 0.167, $\eta_{p}^{2}$ = 0.051). In the S2 rectangular area, there was a significant main effect of time (*F*_(1,37)_ = 21.759, *p* < 0.001,$\eta_{p}^{2}$ = 0.370), but not stimulation (*F*_(1,37)_ = 1.130, *p* = 0.295, $\eta_{p}^{2}$ = 0.030; Figure 4B). Moreover, there was no significant interaction between time and stimulation (*F*_(1,37)_ = 2.196, *p* = 0.147, $\eta_{p}^{2}$ = 0.056).

### Effects of tDCS on the Reach and Manipulation Times

Two-way ANOVA showed that there was no significant interaction between time or stimulation for ether the S1 or S2 of the reach (*F*_(1,37)_ < 0.514, *p* > 0.478, $\eta_{p}^{2}$ < 0.014; Figure 4C) or manipulation times (*F*_(1,37)_ < 0.865, *p* > 0.358, $\eta_{p}^{2}$ < 0.023; Figure 4D). Moreover, there was a significant main effect of time for both the S1 and S2 of the reach (*F*_(1,37)_ > 20.514, *p* < 0.001, $\eta_{p}^{2}$ > 0.357; Figure 4C) and manipulation times (*F*_(1,37)_ > 5.507, *p* < 0.024, $\eta_{p}^{2}$ > 0.130; Figure 4C), but not stimulation in the reach (*F*_(1,37)_ < 0.598, *p* > 0.444, $\eta_{p}^{2}$ < 0.016; Figure 4C) or manipulation times (*F*_(1,37)_ < 1.104, *p* > 0.300, $\eta_{p}^{2}$ < 0.029; Figure 4C).

## Discussion

The present study investigated the effect of atDCS on the left DLPFC on cognitive function, complementary to motor execution. Our results demonstrated that the trajectory in the S2 manual dexterity task requiring decision making was significantly decreased between real and sham tDCS conditions. A previous study reported that the supervisory attention system (SAS) is activated in the PFC during a sequential task that requires attention (Cooper, 2002; Niki et al., 2019). Furthermore, the bilateral DLPFC is activated during Stroop tasks (Adleman et al., 2002). The purpose of the present study was to investigate the modulating effect of atDCS on the left DLPFC on the performance of a sequential decision-making task. Based on these reports, we used the modified Stroop task, which requires cognitive judgment, as the sequential task for the S2 segment. As a result, the trajectory in the S2 segment significantly decreased. Our present results indicate that atDCS on the left DLPFC can facilitate the learning of the SAS component of the sequential task.

A previous study reported that tDCS of the left DLPFC modulates the results of a cognitive task that is competitive with those of the manual dexterity task (Ljubisavljevic et al., 2019). Our results are consistent with those of previous studies, implying that atDCS on the left DLPFC improves cognitive function in a task wherein motor and cognitive functions are complementary, similar to a cognitive task in competitive motor execution. In another study, atDCS to F3 (left DLPFC) improved working memory performance, while cathodal tDCS led to decreases (Zaehle et al., 2011). These reports indicate that atDCS on the left DLPFC improves performance on cognitive tasks, suggesting that the significant decrease in trajectory in our present study was due to improved cognitive function.

Our findings showed a main effect of time on trajectory length, reach time, and manual dexterity task time in S1 and S2, and rectangular area in S2. These results indicate that the task used in the present study encouraged learning progression with task repetition. Furthermore, the trajectory length of S2 decreased more substantially in the real tDCS group than in the sham group, indicating that tDCS had a greater learning enhancement effect compared to baseline in the sequential task.

Moreover, there were no significant differences between the tDCS groups in S1. The task used in the S1 segment was a manual dexterity task that consisted of picking up a peg, which is a routine task and is performed by the basal ganglia and primary motor cortex (Cooper, 2002; Niki et al., 2019). In addition, atDCS has been found to induce modulation of the manual dexterity task by stimulating the primary motor cortex (Parikh and Cole, 2014). These studies suggest that the trajectory length of the S1 did not change in the present study because the stimulation was applied to the DLPFC. In addition, we found no significant differences between groups in the number of trials during the tDCS session, suggesting that the amount of exercise was not a factor in the significance of the S2 trajectory length between the tDCS groups.

All participants in the present study were right-handed with a score of 50 or higher on the Edinburgh Handedness Inventory, and the task was performed with the non-dominant hand. Previous studies have reported that ipsilateral M1 (Kim et al., 1993), and extensive frontal and temporal regions (Grafton et al., 2002) are activated in response to non-dominant hand movements. Furthermore, atDCS has been reported to enhance fine and gross motor hand function only in conditions in which the M1 representing the non-dominant hand was stimulated (Boggio et al., 2006). These reports may indicate that the non-dominant hand tasks used in the present study are more likely to produce task-induced changes in participants’ cortical activity and modulation by the tDCS.

One of the limitations of the present study is that the participants’ awareness of the tDCS condition was significantly different between the tDCS groups, which may have biased the results. For the sham tDCS group, the percentage of correct answers reached up to 90% (18 out of 20). If participants who felt that they were assigned to the sham group intentionally decreased their task performance after tDCS stimulation, it is possible that there was an increase in trajectory length, rectangular area, reach time, and manual dexterity task time in the sham group after stimulation compared with before stimulation; however, the results of the present study showed a main effect of time on trajectory length, reach time, and manual dexterity task time in S1 and S2, and rectangular area in S2. Therefore, it is unlikely that participants in the sham group intentionally performed the task poorly after recognizing that they were in the sham group. Thus, although the participants’ awareness of the condition was significant between the tDCS groups, it is unlikely that there was bias in the results. In addition, the electrode arrangement of tDCS in the present study was only the left DLPFC as the anode and the supraorbital area as the cathode, and the reversal effect of cathodal tDCS was not confirmed (Heeren et al., 2015). Furthermore, we did not verify the placement of electrodes on M1. Hence, additional verification is necessary for future studies. It has been reported that nicotine intake in smoking activates nicotinic acetylcholine receptors and modulates cortical excitability (Grundey et al., 2013). Participants in the present study were not asked about their smoking habits, and this could affect the results. Moreover, the session time of day was not controlled according to participants’ preferences (Salehinejad et al., 2021b). Therefore, the possibility of bias in our results cannot be excluded.

In conclusion, we constructed a new task in which cognitive decisions are reflected in motor actions and investigated whether the performance of the task can be improved by atDCS on the left DLPFC. We set up two components of the task: manual dexterity task only and selection of actions based on the results of cognitive decisions and showed that a single atDCS session on the left DLPFC improved the performance of cognitive tasks complementary to motor execution. In daily life, we perform a variety of sequential tasks while making cognitive decisions to achieve behavioral goals. By elucidating the modulating effect of tDCS on cognitive functions related to motor execution, these results may be used to improve the performance of rehabilitation patients in the future. Further studies are needed to validate the effectiveness of the stimuli used in this study for patients with executive dysfunction.

## Data Availability Statement

The original contributions presented in the study are included in the article, further inquiries can be directed to the corresponding author.

## Ethics Statement

The studies involving human participants were reviewed and approved by the Ibaraki Prefectural University of Health Sciences Review Board. The patients/participants provided their written informed consent to participate in this study. Written informed consent was obtained from the individual(s) for the publication of any potentially identifiable images or data included in this article.

## Author Contributions

Conceptualization: SY, DI, KI, and YK. Methodology and investigation, formal analysis: SY and DI. Writing of the original draft: SY. Review and editing of the manuscript: DI, KI, and YK. All authors contributed to the article and approved the submitted version.

## Conflict of Interest

The authors declare that the research was conducted in the absence of any commercial or financial relationships that could be construed as a potential conflict of interest.

## Publisher’s Note

All claims expressed in this article are solely those of the authors and do not necessarily represent those of their affiliated organizations, or those of the publisher, the editors and the reviewers. Any product that may be evaluated in this article, or claim that may be made by its manufacturer, is not guaranteed or endorsed by the publisher.
